# Cognitive dynamics in preclinical and very mild Alzheimer disease

**DOI:** 10.1002/alz.084213

**Published:** 2025-01-03

**Authors:** Andrew J. Aschenbrenner, Matthew S. Welhaf, Jason J. Hassenstab, Joshua J. Jackson

**Affiliations:** ^1^ Washington University in St. Louis, St. Louis, MO USA

## Abstract

The widespread use of remote digital assessment technology has made it possible to obtain high‐frequency measurements of cognitive function in naturalistic settings (i.e., outside of the clinic). Measurement burst designs feature multiple waves of testing (e.g., multiple tests per day for several weeks) which are repeated at regular intervals (e.g., annually) and have been shown to be feasible, well‐tolerated by participants, and provide substantially more reliable estimates of mean performance than standard assessments. Measurement burst designs also afford an examination of cognitive variability, or the degree to which cognitive scores vary across repeated assessments. Variability can be measured at different time scales including momentary variability across trials within a task, or over different days. Measures of “contextual factors” such as perceived stress, sleep quality, mood, and personality traits are easy to add as well to test potential mechanisms for differences in cognitive variability. An integration of cognitive and contextual variability across multiple time scales provides a more complete picture of change in early‐stage disease and is particularly applicable to preclinical Alzheimer disease (AD) where cognitive declines are extremely subtle and heterogeneous.

Using data from two high‐frequency assessment studies in which cognitively healthy and very mildly impaired older adults completed multiple daily cognitive assessments of episodic memory, working memory, attention, and processing speed for 1 to 3 weeks, we will examine how variability at both short (from trial to trial within a task) and longer (from day to day) timescales change as a function of preclinical AD. We examine potential mechanisms for cognitive variability including increased mind wandering, daily motivation, and variations in daily personality traits. Finally, we will discuss the utility of advanced statistical modeling techniques (e.g., mixed effects location scale models) to answer questions about cognitive variability, and we will examine how our understanding might be further enhanced by employing cognitive process models (such as the diffusion model) that incorporate both accuracy and response time to provide a more complete picture of daily cognitive fluctuations in AD. We end with practical considerations regarding administration of measurement bursts and pose additional questions that will stimulate additional research.

